# MicroRNA-375 restrains the progression of lung squamous cell carcinoma by modulating the ERK pathway via UBE3A-mediated DUSP1 degradation

**DOI:** 10.1038/s41420-023-01499-7

**Published:** 2023-06-29

**Authors:** Junqing Gan, Yu Zhang, Shan Liu, Guannan Mu, Juan Zhao, Wei Jiang, Jiade Li, Qi Li, Yangjiazi Wu, Xinling Wang, Dehai Che, Xiaomei Li, Xiaoyi Huang, Qingwei Meng

**Affiliations:** 1 Department of Medical Oncology, Harbin Medical University Cancer Hospital, 150081 Harbin, Heilongjiang China; 2 Biotherapy Center, Harbin Medical University Cancer Hospital, 150081 Harbin, Heilongjiang China; 3 Department of Pathology, Harbin Medical University Cancer Hospital, 150081 Harbin, Heilongjiang China; 4 NHC Key Laboratory of Cell Transplantation, The First Affiliated Hospital of Harbin Medical University, 150001 Harbin, China

**Keywords:** Non-small-cell lung cancer, miRNAs, Ubiquitylation, Cell growth, Cell migration

## Abstract

MiRNA-375 has been reported to play critical roles in a variety of cancers. To unravel its biological roles, especially its specific mechanisms of action in lung squamous cell carcinoma (LUSC), LUSC tissue microarrays and miRNAscope were performed to identify the miR-375 expression. Associations with clinicopathologic features, survival, and the prognostic value of miR-375 in LUSC were clarified in a retrospective study of 90 pairs of LUSC tissues. In vitro and in vivo gain- and loss-of-function assays were conducted to validate the effects and mechanism of miR-375 in LUSC. The mechanism responsible for interactions was verified by dual-luciferase reporter gene assay, immunoprecipitation (IP) analysis, immunofluorescence (IF) assay and ubiquitination assay. We found that miR-375 had higher expression in noncancerous adjacent tissues than in LUSC tissues. Clinicopathologic analyses showed that miR-375 expression was correlated with pathologic stage and was an independent predictor of overall survival (OS) for LUSC. MiR-375, as a tumor inhibitor, inhibited proliferation and metastasis while promoting apoptosis of LUSC cells. Mechanistic research indicated that miR-375 targeted ubiquitin-protein ligase E3A (UBE3A), which in turn promoted the activity of the ERK signaling pathway via ubiquitin-mediated dual-specificity protein phosphatase 1 (DUSP1) degradation. Collectively, we propose a novel mechanism of tumorigenesis and metastasis of LUSC via the miR-375/UBE3A/DUSP1/ERK axis, which could potentially facilitate new strategies for the treatment of LUSC.

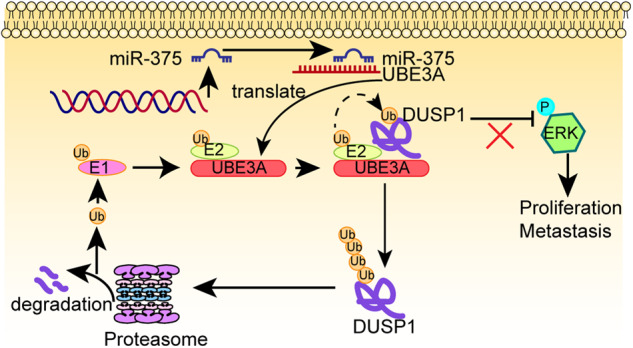

## Introduction

Lung cancer, a lethal malignancy, ranks second in incidence and first in mortality worldwide [1], and lung squamous cell carcinoma (LUSC) represents a common subset of non-small cell lung cancer [2, 3] and accounts for ~30% of lung cancers [4]. LUSC often shows a poor prognosis compared to lung adenocarcinoma (LUAD) owing to a low rate of EGFR gene mutations and ALK fusion genes [5], the lack of available targeted drugs [6], strong tumor heterogeneity and immune responses and low sensitivity to chemotherapy [7]. Consequently, potential therapeutic targets for LUSC treatment are still needed.

MicroRNAs (miRNAs) are small noncoding RNAs with sizes of ~22 nucleotides that play potent roles in the post-transcriptional regulation of gene expression [8]. Insights into the roles of miRNAs in tumorigenesis and progression have identified miRNAs as binding molecules and targets for new therapeutic approaches [9]. Considering the heterogeneous origin of different cancer types, a miRNA can act as either a tumor inhibitor or an oncomiR in cancers, making it indispensable to investigate the exact functions of some specific miRNAs in different cancers in depth. In terms of miR-375, our previous study demonstrated that it was overexpressed in castration-resistant prostate cancer-derived exosomes compared to castration-sensitive prostate cancer-derived exosomes, and the elevated expression was associated with poor prognosis [10]. Moreover, we reported that upregulated expression of miR-375 promoted the progression of DU145 and PC-3 cells [11]. In addition, Tang et al. verified that ectopic expression of miR-375 triggered cell growth and suppressed apoptosis of breast cancer cells [12]. Xu et al. showed that miR-375 could target YAP1 and SP1 to inhibit tumorigenesis and increase sensitivity to chemotherapy in colorectal cancer [13]. These conflicting observations strongly suggest the need for a tissue- and cancer-specific investigation, as in LUSC, to fully reveal the functional portrait of miR-375.

The ubiquitination process involves three factors: E1 ubiquitin-activating enzymes (E1), E2 ubiquitin-conjugating enzymes (E2) and E3 ubiquitin ligases (E3), which confer substrate specificity [14]. Ubiquitin-protein ligase E3A (UBE3A, also known as E6-associated protein [E6AP]), an E3 ubiquitin ligase, is best known for its role in the degradation of P53 in human papillomavirus (HPV)-mediated cancers [15], but growing evidence suggests that UBE3A also contributes to nonviral-related cancers [16]. For example, UBE3A degrades ZNF185 to activate the NOTCH pathway [17]; UBE3A targets SIRT6 in an ANXA2-dependent manner, leading to tumorigenesis of liver cancer [18]. Nevertheless, little is known about the role of UBE3A in LUSC.

In this study, we showed that miR-375 had low expression in cells of LUSC tissues whose proliferation and metastasis could be substantially inhibited by ectopic expression of miR-375. Mechanistically, miR-375 stabilizes the extracellular signal-regulated protein kinase (ERK) suppressor dual-specificity protein phosphatase 1 (DUSP1) by targeting UBE3A. Thus, miR-375 acts as a tumor suppressor gene, and modulation of miR-375 expression might be a promising therapeutic strategy to treat LUSC.

## Results

### MiR-375 was downregulated in LUSC tissues and associated with a good prognosis

**Fig. 1:**
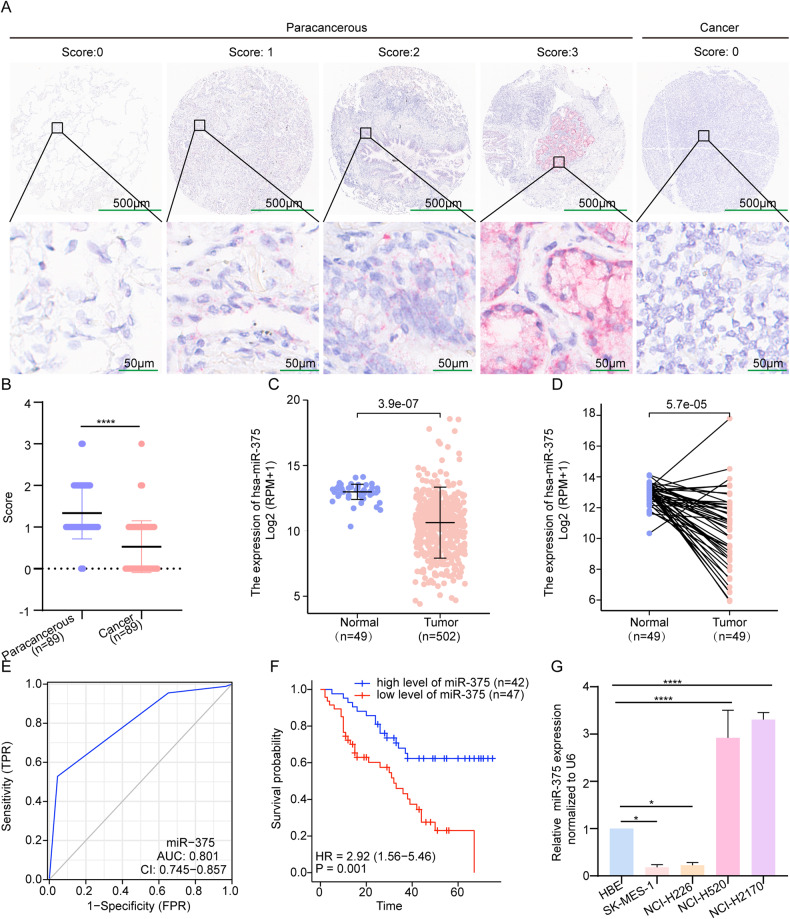
Downregulation of miR-375 was associated with poor prognosis in LUSC. **A** Representative pictures of the RNAscope assay showing the expression of miR-375 in cancer and paracarcinoma tissues, magnification ×40 and ×200, respectively. **B** The scores of miR-375 expression in cancer and paracarcinoma tissues. **C**, **D** MiR-375 expression levels in unpaired (**C**) and paired (**D**) LUSC tissues (*n* = 502) and normal tissues (*n* = 49) from TCGA. **E** The ROC curve distinguished normal from LUSC tumor tissue, with an AUC of 0.801. **F** Relationship between miR-375 expression and OS was assessed by a Kaplan–Meier plot. The log-rank test was conducted to indicate statistically significant differences among the survival curves. **G** qRT-PCR of the expression of miR-375 in four LUSC cell lines compared to that in HBE cells. Amplification of U6 served as an internal control.

To verify the clinical significance of miR-375, we used a tissue microarray consisting of 90 pairs of LUSC and adjacent noncancerous lung tissues for MiRNAscope analysis. Except for one tissue that was dissociated from the slide during the experimental procedure, 89 pairs of tissues were successfully stained and evaluated. Figure 1A, B demonstrates that miR-375 was significantly downregulated in LUSC tissues, which was consistent with the data obtained from the TCGA database (Fig. 1C, D). As summarized in Table 1, the reduction in miR-375 expression was positively linked to pathologic stage (*P* < 0.001), whereas miR-375 expression was not significantly related to tumor size, lymph node metastasis, distal metastasis, clinical stage, sex, or age. This finding may be due to the limited sample size and the sampling bias that only resectable tumors were included in this study, as evidenced by only one patient with distal metastasis on the tissue array (Table 1). Receiver operating characteristic (ROC) analyses were performed to validate the area under the ROC curve (AUC) and identify optimal cutoff values. As illustrated in Fig. 1E, the AUC for this ROC curve was 0.801, and the best cutoff value was 0.5, according to which the LUSC patients were substratified into low and high expression groups. Next, we detected whether the expression of miR-375 in LUSC was related to prognosis. The Kaplan‒Meier curve indicated good overall survival for patients with high levels of miR-375 (Fig. 1F, *P* = 0.001). In addition, univariable and multivariate regression analyses both suggested that low miR-375 expression was an independent prognostic factor for poor survival in LUSC (Table 2). In summary, our data clarified that miR-375 was poorly expressed in LUSC and closely related to the clinicopathological features and prognosis of LUSC.

**Table 1:** Clinocopathologic characteristics of LUSC patients stratified by miR-375 expression.

Characteristic	miR-375 expression		*P*
	High	Low	
*n*	42	47	
T stage, *n* (%)			0.772
T1	1 (1.6%)	1 (1.6%)	
T2	19 (31.1%)	14 (23%)	
T3	10 (16.4%)	10 (16.4%)	
T4	2 (3.3%)	4 (6.6%)	
N stage, *n* (%)			0.544
N0	24 (30.8%)	20 (25.6%)	
N1	12 (15.4%)	17 (21.8%)	
N2	2 (2.6%)	3 (3.8%)	
M stage, *n* (%)			0.472
M0	41 (46.1%)	47 (52.8%)	
M1	1 (1.1%)	0 (0%)	
Pathologic stage, *n* (%)			**<0.001**
I	3 (3.4%)	0 (0%)	
II	37 (41.6%)	26 (29.2%)	
III	2 (2.2%)	21 (23.6%)	
Clinical stage, *n* (%)			0.421
1	10 (16.4%)	6 (9.8%)	
2	14 (23%)	12 (19.7%)	
3	7 (11.5%)	11 (18%)	
4	1 (1.6%)	0 (0%)	
Sex, *n* (%)			0.600
Female	2 (2.2%)	1 (1.1%)	
Male	40 (44.9%)	46 (51.7%)	
Age, mean ± SD	61.52 ± 9.04	63.55 ± 8.52	0.279

Note: One patient was excluded due to technical failure during RNAScope procedure.

Bold values indicates statistical significant *P* values (*P* < 0.05).

**Table 2:** The univariable and multivariable cox regression analysis of overall survival (OS) in LUSC patients.

Characteristics	Total (*N*)	Univariate analysis		Multivariate analysis	
		Hazard ratio (95% CI)	*P* value	Hazard ratio (95% CI)	*P* value
T stage	61		**0.005**		
T1	2	Reference			
T2	33	0.718 (0.092–5.593)	0.752	1.064 (0.126–9.016)	0.954
T3	20	1.350 (0.174–10.481)	0.774	0.940 (0.097–9.091)	0.958
T4	6	4.781 (0.565–40.478)	0.151	1.762 (0.150–20.698)	0.652
N stage	78		0.130		
N0	44	Reference			
N1	29	1.652 (0.864–3.158)	0.129		
N2	5	2.565 (0.870–7.565)	0.088		
M stage	89		0.286		
M0	88	Reference			
M1	1	2.970 (0.402–21.938)	0.286		
Pathologic stage	89		0.280		
I	3	Reference			
II	63	1.810 (0.247–13.289)	0.559		
III	23	2.865 (0.376–21.849)	0.310		
Clinical stage	61		**0.001**		
1	16	Reference			
2	26	1.347 (0.414–4.377)	0.621	1.271 (0.360–4.491)	0.710
3	18	5.520 (1.801–16.913)	**0.003**	4.523 (0.958–21.347)	0.057
4	1	7.499 (0.806–69.782)	0.077	7.960 (0.531–119.282)	0.13
Age	89	1.024 (0.989–1.059)	0.177		
Sex	89		0.688		
Male	86	Reference			
Female	3	1.339 (0.323–5.558)	0.688		
miR-375 expression	89		**<0.001**		
High	42	Reference			
Low	47	2.919 (1.560–5.461)	**<0.001**	3.079 (1.355–6.996)	**0.007**

Bold values indicates statistical significant *P* values (*P* < 0.05).

To screen suitable LUSC cells for subsequent investigation, we determined the expression of miR-375 in normal pulmonary epithelial HBE cells and 4 LUSC cell lines. Compared to HBE cells, NCI-H2170 and NCI-H520 cells expressed higher levels, while SK-MES-1 and NCI-H226 cells expressed lower levels of miR-375 (Fig. 1G).

### MiR-375 inhibits the proliferation, migration, and invasion and promotes apoptosis of LUSC cells in vitro

**Fig. 2:**
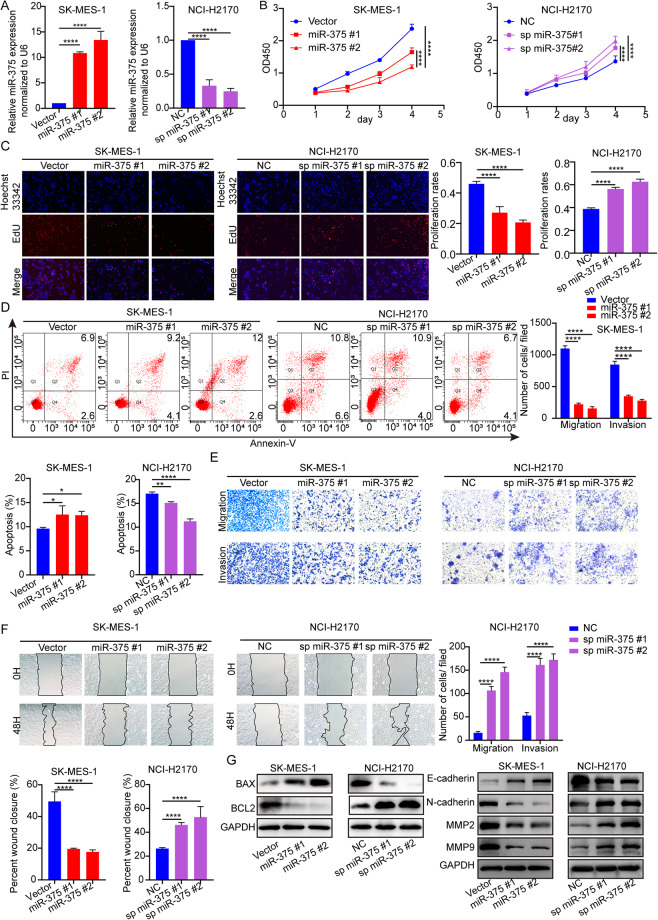
Upregulation of miR-375 retards LUSC proliferation and metastasis and accelerates apoptosis in vitro. **A** The efficiency of miR-375 overexpression in SK-MES-1 cells and inhibition in NCI-H2170 cells was determined by qRT‒PCR. Amplification of U6 served as an internal control. **B** A CCK-8 assay was used to determine cell viability at the indicated time points in SK-MES-1 cells stably overexpressing miR-375 and NCI-H2170 cells with stable silencing of miR-375. **C** The proliferation of SK-MES-1 or NCI-H2170 cells after overexpression or silencing of miR-375 examined by EdU assays, magnification ×100. **D** Flow cytometry analyses of cell apoptosis in control and SK-MES-1 cells with miR-375 overexpression or NCI-H2170 cells with miR-375 silencing. **E** Transwell assay and **F** wound-healing assay indicating the effects of miR-375 overexpression or suppression on LUSC cell migration and invasion, magnification ×100. **G** Protein expression of BAX, BCL2, E-cadherin, N-cadherin, MMP2, and MMP9 was tested by western blots. GAPDH was used as a loading control. Vector: SK-MES-1 cell colony transfected with pSUPER-RETRO-Puro-empty vector. MiR-375#1: cell colony 1 transfected with pSUPER-RETRO-Puro-miR-375 recombinant vector. MiR-375#2: cell colony 2 transfected with pSUPER-RETRO-Puro-miR-375 recombinant vector. NC: NCI-H2170 cell colony transfected with pHB-U6-MCS-PGK-PURO-empty vector. Sp miR-375#1: cell colony 1 transfected with pHB-U6-MCS-PGK-PURO-miR-375 sponge vector. Sp miR-375#2: cell colony 2 transfected with pHB-U6-MCS-PGK-PURO-miR-375 sponge vector.

To gain insight into the potential role of miR-375, we overexpressed miR-375 in SK-MES-1 cells and knocked down miR-375 in NCI-H2170 cells. QRT‒PCR was conducted to evaluate the overexpression and inhibition efficiency among the stably transfected cell colonies (Fig. 2A). CCK-8 and EdU assays were conducted to determine cell growth. As shown in Fig. 2B, miR-375 overexpression strikingly reduced LUSC cell proliferation, while miR-375 knockdown increased LUSC cell proliferation, which was further verified by EdU assays (Fig. 2C). Ectopically expressed miR-375 augmented the apoptosis rate, and miR-375 knockdown led to the opposite effect (Fig. 2D). To clarify the effect of miR-375 on cell migration and invasion, we performed transwell and wound-healing assays. As expected, miR-375 overexpression retarded cell invasion and migration, while miR-375 depletion elicited the opposite results in LUSC cells (Fig. 2E, F). Additionally, western blotting indicated that BAX and E-cadherin levels increased while the expression levels of BCL2, MMP2, MMP9, and N-cadherin decreased as miR-375 was overexpressed, along with an opposite expression pattern in the same markers upon miR-375 silencing (Fig. 2G).

### UBE3A is targeted by miR-375

**Fig. 3:**
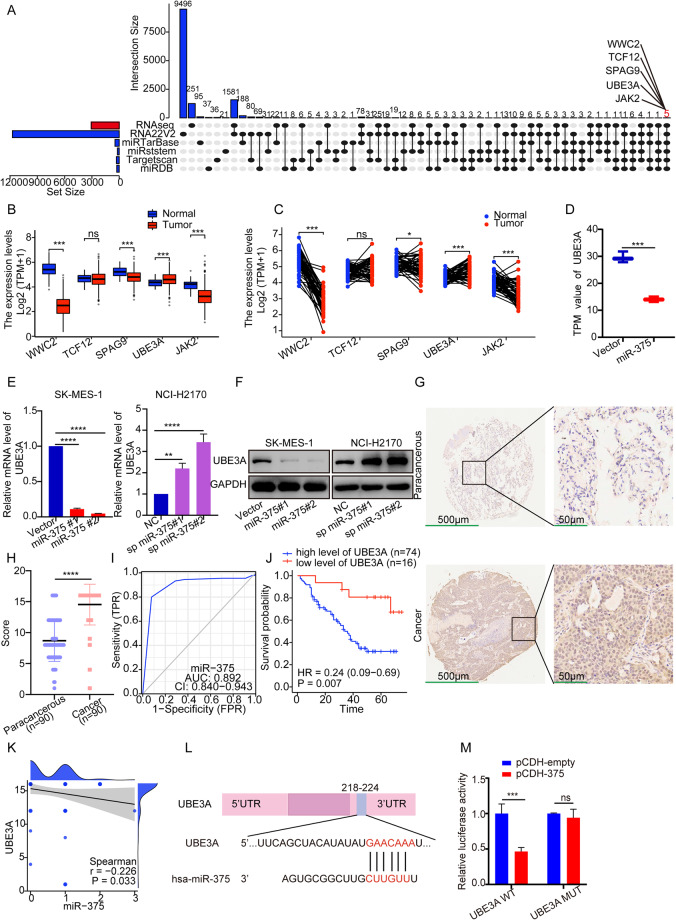
UBE3A is a target of miR-375. **A** Overlaps of miR-375 predicted targets from 5 public databases and the differentially expressed genes determined by RNA sequencing after miR-375 was overexpressed in SK-MES-1 cells, and five genes were selected. **B**, **C** The expression of five selected genes in unpaired (**B**) and paired (**C**) LUSC (*n* = 502) and normal tissues (*n* = 49) from TCGA. **D** RNA-seq, **E** qRT‒PCR and **F** western blotting were carried out to verify the relationship between miR-375 and UBE3A. GAPDH served as an internal control for the detection of UBE3A mRNA. **G** IHC assay displaying UBE3A expression between cancer and adjacent normal tissues, with magnifications of ×40 and ×200, respectively. **H** Quantitative immunohistochemistry analysis of UBE3A in LUSC and paracarcinoma tissues. **I** ROC curve analysis for differentiating normal from tumor tissue, with an AUC of 0.892. **J** Relationship between UBE3A expression and OS was assessed by a Kaplan–Meier plot. The log-rank test was conducted to explore statistical significance based on the survival curves. **K** Correlation between UBE3A and miR-375 expression levels in LUSC tissues (*n* = 89 patients). **L** The putative binding sites of miR-375 within the 3’UTR of UBE3A mRNA predicted by the TargetScan database. **M** Luciferase reporter assays were carried out in HEK293T cells cotransfected with miR-375 expression vector or control vector with the luciferase reporter plasmid containing either wild‐type (WT) or mutant (MUT) 3′UTR of UBE3A to confirm the regulation of UBE3A by miR-375.

Since miR-375 modulated the proliferation, apoptosis, and migration of NCI-H2170 and SK-MES-1 cells, we aimed to explore the underlying molecular mechanisms in LUSC. RNA-seq results showed that a total of 3074 genes were differentially expressed in the miR-375-overexpressing SK-MES-1 cells compared to the controls (Supplementary Table S2). Further analysis demonstrated a significant enrichment of GO terms associated with negative regulation of cellular processes and cell cycle processes (Supplementary Fig. S1A–C). The KEGG analysis showed that miR-375 was mainly associated with herpes simplex virus 1 infection and the TNF signaling pathway (Supplementary Fig. S1D). To identify putative targets of miR-375, we drew an UpSet plot illustrating shared genes in five datasets and our RNA-seq readouts (*P* adj<0.01 and fold changeå 1.5) and identified 5 genes: WWC2, TCF12, SPAG9, UBE3A, and JAK2 (Fig. 3A), among which only UBE3A was upregulated in LUSC tissues compared to paired and unpaired normal lung tissues from TCGA database (Fig. 3B, C). Hence, UBE3A was selected for subsequent validation. According to our mRNA sequencing, the UBE3A mRNA level was reduced in SK-MES-1 cells upon miR-375 overexpression (Fig. 3D). Moreover, qRT‒PCR and western blotting revealed that forced expression of miR-375 resulted in reduced UBE3A at both the mRNA and protein levels and vice versa (Fig. 3E, F). Importantly, the IHC assay reversely corroborated the signature of UBE3A in that UBE3A, localized in both the nucleus and cytoplasm of LUSC cells, was overexpressed in LUSC tissues compared to adjacent nontumor lung tissues (Fig. 3G, H). A high level of UBE3A possessed strong potential (AUC = 0.892) to distinguish LUSC patients from healthy subjects (Fig. 3I). Next, we selected the optimal cutoff based on the point on the ROC curve that was farthest from a chance result to divide patients into high- and low-expression groups. As shown in Fig. 3J, the overall survival was significantly prolonged in the UBE3A-low expression group. Concurrently, our correlation analysis demonstrated that miR-375 was negatively correlated with UBE3A expression (Spearman *r* = -0.226, *P* = 0.033, Fig. 3K). To verify the physical interaction between miR-375 and the mRNA of UBE3A, we cloned the 3′UTRs of UBE3A containing the mutant and wild-type putative binding sites, displayed in Fig. 3L, into the P-MIR-Report firefly luciferase vector. As shown in Fig. 3M, the luciferase reporter assays showed a significant reduction in luciferase activity as p-MIR-wt UBE3A-3′UTR was cotransfected with the vector harboring the miR-375 coding sequence, while there was no significant change in the transfection of the p-MIR-mut UBE3A-3′UTR vector along with the miR-375 expression vector. These findings collectively suggested that UBE3A was a direct target of miR-375.

To understand the mechanisms by which UBE3A mediates LUSC tumorigenesis, we utilized the STRING Protein‒Protein Interaction database to predict the proteins that might interact with UBE3A [19] and obtained 50 candidates (Supplementary Fig. S2A), which were used for GO and KEGG analysis. According to the GO analysis (Supplementary Table S6), cellular functions such as protein stabilization, tight junction, and proteasome binding were related to these candidates. In parallel, the KEGG enrichment revealed the relationship between these genes and human papillomavirus infection, ubiquitin-mediated proteolysis, pathways of neurodegeneration-multiple diseases, the p53 signaling pathway and Alzheimer’s disease (Supplementary Table S6), consistent with what has been reported in the literature [16, 20–23].

### UBE3A is essential for miR-375-mediated attenuation of proliferation and metastasis by inactivating the ERK pathway

**Fig. 4:**
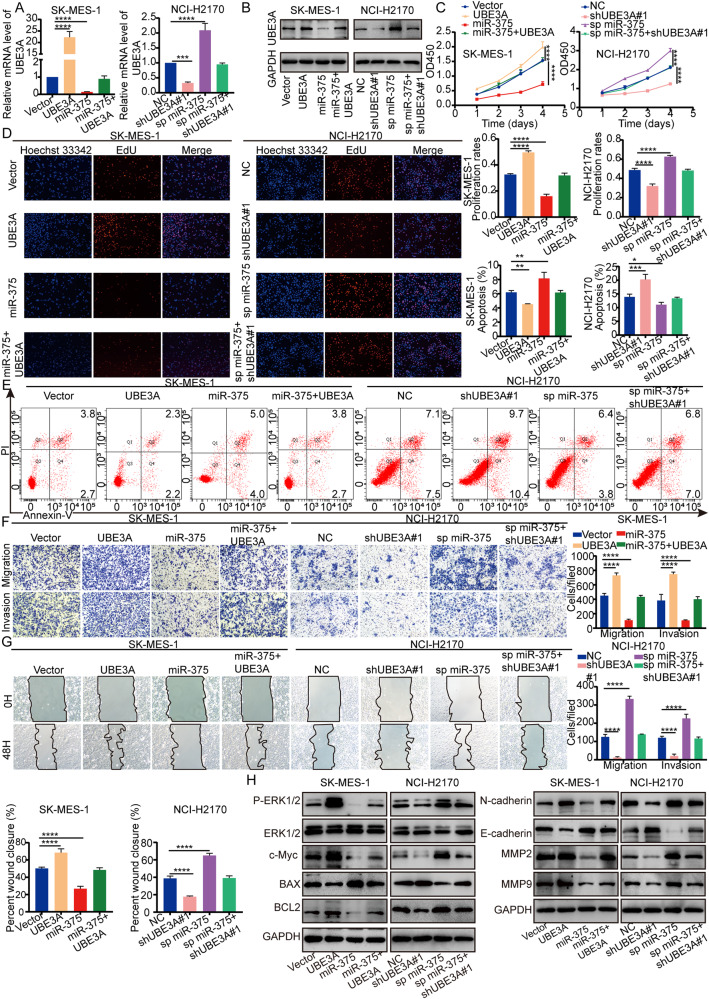
UBE3A is required for miR-375-induced inhibition of the malignant phenotype of LUSC by inactivating the ERK pathway. **A** qRT‒PCR and **B** western blot showing the mRNA and protein levels of UBE3A in SK-MES-1 cells transfected with UBE3A and/or miR-375 expression vector(s) and in NCI-H2170 cells transfected with shUBE3A#1 and/or sp miR-375 vector(s). **C** A CCK-8 assay was used to determine the cell viability at the indicated time points in SK-MES-1 cells transfected with UBE3A and/or miR-375 expression vector(s) and in NCI-H2170 cells transfected with shUBE3A#1 and/or sp miR-375 vector(s). **D** The proliferation of SK-MES-1 cells transfected with UBE3A and/or miR-375 expression vector(s) and NCI-H2170 cells transfected with shUBE3A#1 and/or sp miR-375 vector(s) was determined by EdU assay, magnification ×100. **E** Flow cytometry analyses of cell apoptosis in SK-MES-1 cells transfected with UBE3A and/or miR-375 expression vector(s) and in NCI-H2170 cells transfected with shUBE3A#1 and/or sp miR-375 vector(s). **F** Transwell assay and **G** wound-healing assay were used to test the migration and invasion of SK-MES-1 cells in response to overexpression of miR-375 and/or UBE3A and NCI-H2170 in response to suppression of miR-375 and/or UBE3A, magnification ×100. **H** Protein expression of p-ERK1/2, c-Myc, BAX, BCL2, E-cadherin, N-cadherin, MMP2 and MMP9 was determined by western blots in SK-MES-1 cells transfected with UBE3A and/or miR-375 expression vector(s) and in NCI-H2170 cells transfected with shUBE3A#1 and/or sp miR-375 vector(s). GAPDH was used as a loading control.

Given the above findings, we surmised that miR-375 restrained the proliferation, migration, and invasion and facilitated the apoptosis of LUSC by targeting UBE3A. For confirmation of this hypothesis, UBE3A expression was upregulated in SK-MES-1 cells transfected with the MCS-PGK-Puro-UBE3A vector and silenced in NCI-H2170 cells transfected with three different shRNAs, with shUEB3A #1 and #3 showing higher inhibitory efficiency (Supplementary Fig. S3A). As shown in Fig. 4A, B and Supplementary Fig. S3B, upregulated UBE3A significantly attenuated the inhibitory effects of miR-375 overexpression on both the mRNA and protein levels of UBE3A in SK-MES-1 cells, and silencing UBE3A attenuated the promotive effects of miR-375 inhibition on both the mRNA and protein levels of UBE3A in NCI-H2170 cells. For proof-of-function experiments, upregulated miR-375 enhanced apoptosis and inhibited proliferation, migration and invasion of SK-MES-1 cells, which could be fully rescued by UBE3A overexpression (Fig. 4C–G). In contrast, the effects of miR-375 inhibition on NCI-H2170 cells were completely rescued by downregulation of UBE3A (Fig. 4C–G and Supplementary Fig. S3C–G). Concurrently, western blotting demonstrated that the expression of apoptotic and migration markers was also rescued when UBE3A expression was enhanced or silenced in the miR-375-overexpressing or miR-375-depleted LUSC cells (Fig. 4H and Supplementary Fig. S3H). To explore the regulatory mechanism of UBE3A in LUSC progression, we used GSEA to explore the potential pathways in which UBE3A might be involved and found that UBE3A was negatively associated with the ERK pathway in HW (Supplementary Fig. S2B). Indeed, overexpression of UBE3A in SK-MES-1 cells reversed the suppressive effect of miR-375 upregulation on p-ERK1/2 expression, whereas inhibition of UBE3A in NCI-H2170 cells reversed the effect of miR-375 knockdown on p-ERK1/2 expression (Fig. 4H and Supplementary Fig. S3H). Therefore, we found that miR-375 hampered LUSC progression by inactivating the ERK pathway by targeting UBE3A.

### MiR-375 inhibits UBE3A-mediated ubiquitination and degradation of DUSP1

**Fig. 5:**
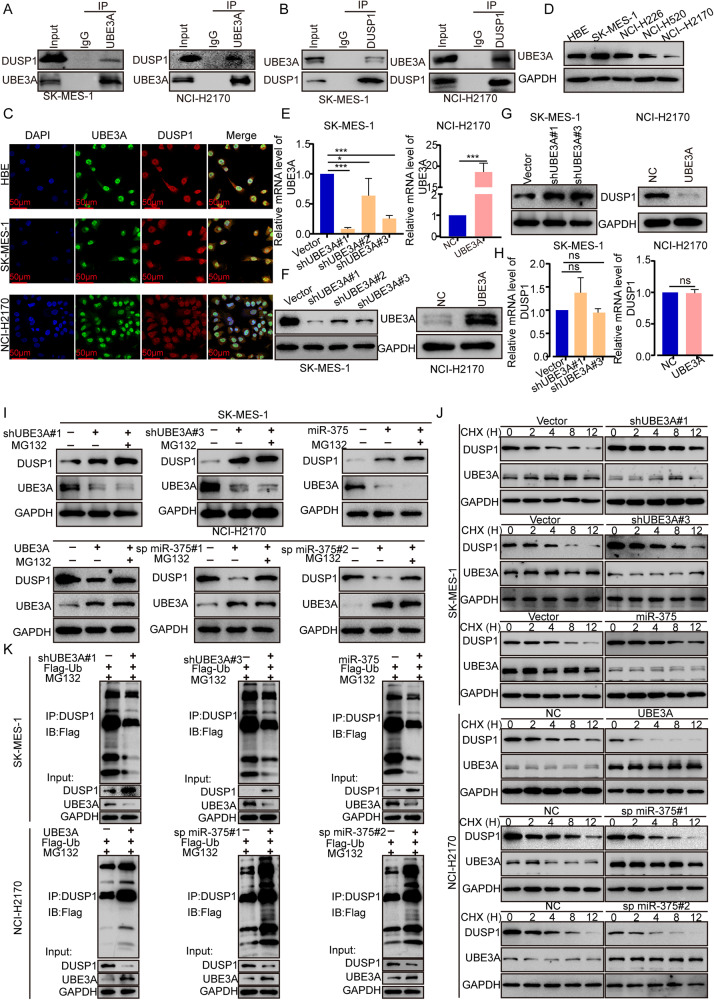
MiR-375 inhibits UBE3A-mediated ubiquitination and degradation of DUSP1. **A**, **B** SK-MES-1 and NCI-H2170 cell lysates were subjected to immunoprecipitation with negative control IgG, **A** anti-UBE3A or **B** anti-DUSP1 antibodies. The immunoprecipitates were then detected using the indicated antibodies. **C** Immunofluorescence analysis was used to detect subcellular colocalization of UBE3A and DUSP1, magnification ×600. **D** Western blotting was conducted to determine the expression of UBE3A in four LUSC cell lines and HBE cells. **E**, **F** The efficiency of UBE3A inhibition in SK-MES-1 cells and overexpression in NCI-H2170 cells, as determined by qRT‒PCR (**E**) and western blotting (**F**). **G**, **H** Western blotting and qRT‒PCR were carried out to verify the protein and mRNA levels of DUSP1 in UBE3A-depleted or UBE3A-overexpressing cell lines. **I** SK-MES-1 and NCI-H2170 cells were transfected with the indicated vector. After 48 h, the cell lysates were subjected to western blot analysis. The selected cells were treated with or without 10 µM MG132 for 12 h before being harvested. **J** SK-MES-1 and NCI-H2170 cells were transfected with the indicated vector. After 48 h, the cells were treated with 100 μg/mL CHX and collected for western blot analysis at the indicated time points. **K** SK-MES-1 and NCI-H2170 cells were transfected with the indicated plasmids. After 48 h, cell lysates were used to test the polyubiquitination level of DUSP1. The selected cells were treated with 10 µM MG132 for 12 h before being harvested.

We next asked how UBE3A modulated p-ERK1/2 expression. DUSP1, a tumor suppressor, was reported to negatively regulate the ERK pathway [24]. Therefore, we speculated that UBE3A might regulate the ERK pathway by decreasing DUSP1 levels. To test this hypothesis, we first validated the interaction between UBE3A and DUSP1 by immunoprecipitating endogenous UBE3A and DUSP1 in SK-MES-1 and NCI-H2170 cells. In both LUSC cell lines, UBE3A coimmunoprecipitated with DUSP1 and vice versa (Fig. 5A, B). As shown in Fig. 5C, the merged IF image displayed a yellow/light yellow signal, indicating the subcellular colocalization of UBE3A and DUSP1, both in the cytoplasm and nucleus, in LUSC cell lines and HBE cells. Next, to ascertain the relationship between UBE3A and DUSP1, we measured the expression of UBE3A in LUSC cell lines (NCI-226, NCI-H520, NCI-H2170 and SK-MES-1) and HBE cells. As Fig. 5D shows, UBE3A expression was downregulated in NCI-H2170 cells but upregulated in SK-MES-1 cells. While the expression of UBE3A was depleted in SK-MES-1 cells or enhanced in NCI-H2170 cells (Fig. 5E, F), the protein expression of DUSP1, which was assessed by western blotting (Fig. 5G), was upregulated, concomitant with the intact mRNA level of DUSP1, as revealed by the qRT‒PCR assay (Fig. 5H). This observation suggested that DUSP1 expression was post-transcriptionally regulated by UBE3A in LUSC.

Given that UBE3A is an E3 ubiquitin ligase [17], DUSP1 could be subjected to ubiquitination [25], and we then asked if UBE3A downregulated DUSP1 by promoting DUSP1 ubiquitination and degradation. To confirm this hypothesis, we used MG132 (a proteasome inhibitor) to treat human LUSC cells. Compared with the DMSO control, MG132 treatment distinctly enhanced UBE3A depletion- or miR-375 overexpression-induced upregulation of DUSP1 in SK-MES-1 cells and rescued UBE3A upregulation or miR-375 inhibition-mediated suppression of DUSP1 in NCI-H2170 cells (Fig. 5I). In addition, the CHX chase experiment indicated that DUSP1 was degraded at a much faster rate in cells with overexpression of UBE3A or inhibition of miR-375 than in control NCI-H2170 cells and was mutually complementary with UBE3A suppression and miR-375 overexpression stabilizing DUSP1 in SK-MES-1 cells (Fig. 5J). Importantly, ubiquitination analysis revealed that UBE3A suppression or miR-375 ectopic overexpression decreased DUSP1 ubiquitination in SK-MES-1 cells, whereas UBE3A overexpression or miR-375 suppression increased DUSP1 ubiquitination in NCI-H2170 cells (Fig. 5K). To gain further insight into the subcelular location for ubiquitination of DUSP1, we performed coimmuniprecipitation assays using cytosolic and nuclear protein fractions. Consistent with IF results, UBE3A coimmunoprecipitated with DUSP1 in both cytosol and nucleus and vice versa (Supplementary Fig. S4A, B). Furthermore, DUSP1 ubiquitination can occur in both cytoplasm and nucleus (Supplementary Fig. S4C). Interestingly, after treatment with leptomycin B (LMB), a drug inhibitor of nuclear export, ubiquitination of DUSP1 was attenuated in cytoplasm while increased in nucleus (Supplementary Fig. S4D). In summary, UBE3A was revealed to participate in DUSP1 ubiquitination and degradation to activate the ERK pathway. Collectively with the finding that ectopic expression of UBE3A partially reversed the miR-375 overexpression-mediated elevation of DUSP1 and vice versa (Supplementary Fig. S4E), it was suggested that miR-375 regulates DUSP1 through UBE3A.

### MiR-375 induces proliferative suppression of LUSC cells in vivo

**Fig. 6:**
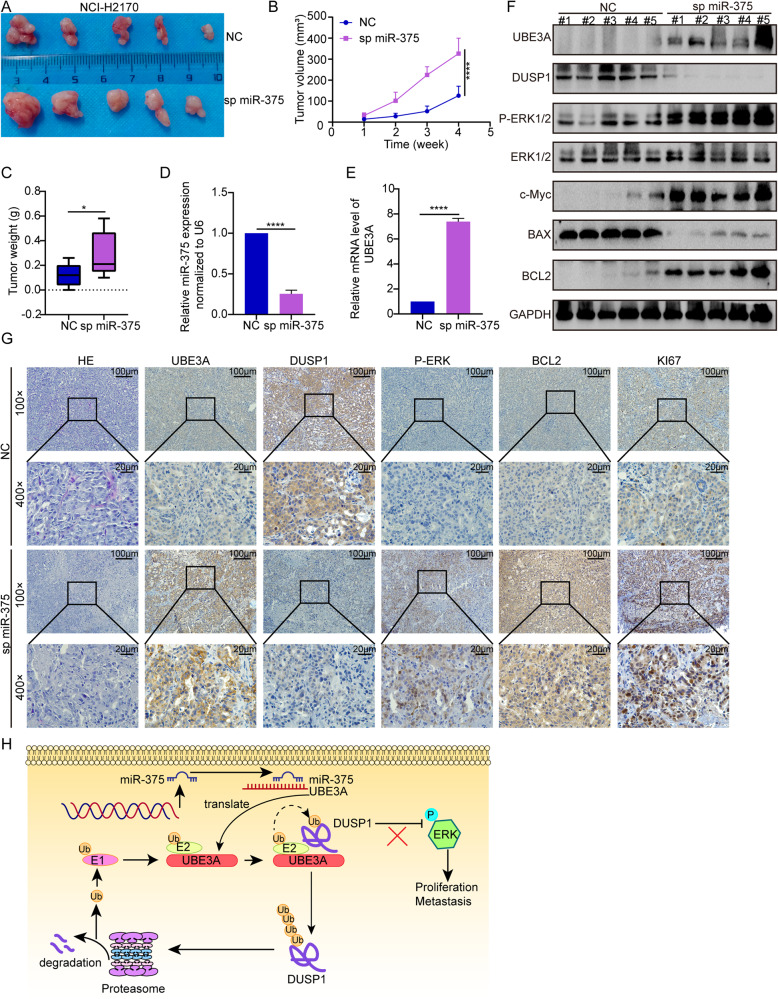
MiR-375 silencing accelerates LUSC tumorigenesis in vivo. **A** Representative images of tumors, **B** tumor volume and **C** tumor weight in nude mice bearing NCI-H2170 with or without miR-375 suppression (*n* = 5). **D**, **E** The expression of miR-375 (**D**) and mRNA level of UBE3A (**E**) in xenograft tumors, determined using qRT-qPCR. **F** Western blots and **G** HE and IHC were employed to analyze the expression of the indicated antibodies. **H** Graphical illustration of the role of miR-375 in regulating LUSC progression.

To evaluate the role of miR-375 in the progression of LUSC in vivo, we established a xenograft tumor model. During the follow-up and at the endpoint, the miR-375-suppressed tumors significantly promoted the growth of NCI-H2170 tumors in terms of tumor size, tumor volume, and tumor weight (Fig. 6A–C), consistent with our in vitro results. In addition, in line with the aforementioned qRT‒PCR results (Fig. 2A), the inhibition of miR-375 expression was sustained in vivo (Fig. 6D). The depletion of miR-375 led to a significant increase in UBE3A levels, as suggested by the qRT‒PCR and western blot results (Fig. 6E, F). Moreover, our western blot results demonstrated that the expression of p-ERK1/2, BCL2 and c-Myc was significantly elevated, while DUSP1 and BAX were decreased, in response to miR-375 depletion (Fig. 6F). Consistently, IHC analysis indicated elevated expression of UBE3A, Ki67, BCL2, and p-ERK1/2, whereas decreased expression of DUSP1 in the miR-375-depleted tumors compared with the NC tumors produced the opposite results (Fig. 6G). Furthermore, the overexpression of miR-375 in SK-MES-1 cells led to an opposite result with the tumor size, tumor volume, tumor weight, and same markers showing altered expression in the opposing direction (Supplementary Fig. S5). All of the results presented above strongly confirmed that miR-375 was a tumor inhibitor for LUSC.

## Discussion

To date, LUSC patients suffer from late-stage diagnosis and poor clinical prognosis as well as a lack of available targeted therapeutics compared to lung adenocarcinoma patients. Clinically feasible biomarkers and accurate targets for the development and progression of LUSC have not been fully investigated [26, 27]. In our study, we identified miR-375 as a potential prognostic marker that is downregulated in LUSC. Patients with low expression of miR-375 had a significantly shorter overall survival than those with high miR-375 expression. Low expression of miR-375 was also found to be positively linked to pathologic stage, and miR-375 could effectively distinguish LUSC tissues from nontumor tissues with an AUC value of 0.801. Collectively, these clinical-derived data strongly indicated that miR-375 might be a crucial factor in the progression of LUSC. Indeed, our study suggested that miR-375 is a pleiotropic modulator of LUSC cells, restraining growth, invasion, and migration and promoting apoptosis of SK-MES-1 and NCI-H2170 cells.

UBE3A (E6AP) is a 100 kDa cellular protein that regulates protein expression through its E3 ligase function that directs proteins to the ubiquitin proteasome system [28]. UBE3A interacted with ENO1 to contribute to its ubiquitination and proteasomal degradation in breast cancer cells [29]. Moreover, the RBPJ/DAPK3/UBE3A/PBRM1/p21 axis induced increased sensitivity of renal cancer cells to CDK4/6 inhibitors [30]. In addition, UBE3A is a transcriptional coactivator of steroid hormone receptors such as estrogen, progesterone, androgen and glucocorticoid receptors [31], partially explaining the nuclear location of ubiquitination pathway-associated enzymes. UBE3A was confirmed to act as a coactivator of the glucocorticoid receptor and simultaneously target the liganded glucocorticoid receptor for proteasomal degradation [32]. Moreover, UBE3A interacted with IRF and aggravated IRF-dependent transcription in a UBE3A-deficient Angelman syndrome mouse model [33]. In our study, we found that UBE3A is a direct target of miR-375. UBE3A was overexpressed in LUSC tissues compared to adjacent nontumor lung tissues and showed a better ability to distinguish healthy subjects from patients. The survival period was clearly prolonged in the UBE3A-low expression group. MiR-375 was negatively correlated with UBE3A levels in LUSC tissues, and importantly, miR-375 inhibited LUSC progression by inactivating the ERK pathway by targeting UBE3A.

Emerging studies have shown that ERK signaling plays an indispensable role in the progression of cancer. For example, FAM163A promotes LUSC cell proliferation by increasing the levels of p-ERK [34]. MiR-1 inhibited the proliferation and chemosensitivity of breast cancer cells by suppressing the MEK/ERK pathway [35]. Silencing LASP2 promoted HepG2 cell viability and migration by upregulating the expression of p-ERK [36]. Therefore, we speculated that deficiencies or downregulation of negative regulators of the ERK signaling pathway could activate the progression of LUSC. DUSP1 (MKP-1), a member of the threonine-tyrosine dual-specificity phosphatase family, is a key negative regulator of the MAPK/ERK pathway [37, 38]. DUSP1 overexpression increased gefitinib sensitivity by inactivating the ERK pathway [39]. Moreover, DUSP1 contributes to CRC cell proliferation and suppresses apoptosis by inactivating MEK/ERK/p38 signaling [40]. Here, we focused on the ubiquitination function of UBE3A and clarified that UBE3A potentiated DUSP1 ubiquitination and degradation to activate the ERK pathway. Notably, in HEK293T cells, DUSP1 ubiquitination is dependent on K48 but not K63, and a mutation of the lysine residue K280 or K289 on DUSP1 induced a notable decrease in DUSP1 polyubiquitination, while a mutation in K230 did not change DUSP1 ubiquitination [41]. As UBE3A-mediated polyubiquitylation was revealed to be K48-linked [18], UBE3A-led DUSP1 ubiquitination may be K48 dependent. In our future in vitro ubiquitylation experiments, this hypothesis will be further validated.

## Conclusion

Our study provides novel insights into the function of miR-375 in LUSC and presents evidence for the molecular mechanism of pleiotropic actions of miR-375. This study identifies miR-375 as the predominant tumor inhibitor in LUSC. Mechanistically, miR-375 targeted UBE3A to prevent UBE3A-induced DUSP1 ubiquitination and degradation and then inactivated the ERK pathway (Fig. 6H). Thus, miR-375 functions to prevent ERK activity in LUSC by targeting the UBE3A/DUSP1 axis, which might be a promising target for LUSC diagnostics and therapeutics.

## Materials and methods

### Cell lines and reagents

Human bronchial epithelial cells (HBE), the LUSC cell lines NCI-H520, NCI-H226, NCI-H2170 and SK-MES-1 (the Institute of Biochemistry and Cell Biology of the Chinese Academy of Sciences, China) and HEK-293 cells were routinely incubated at 37 °C in a 5% CO_2_ humidified atmosphere in RPMI 1640 (Biological Industries, Israel) or MEM (for SK-MES-1, Biological Industries, Israel) or DMEM (for HEK-293, Biological Industries, Israel) with 10% fetal bovine serum (FBS, Biological Industries, Israel) and 1% penicillin/streptomycin (Beyotime Biotechnology, China). Cell lines were authenticated by STR Profiling (ATCC) upon receipt and verified free of mycoplasma contamination (Myco-LumiTM Mycoplasma Detection Kit, Beyotime).

MG132 (MedChemExpress, USA, 133407-82-6) was dissolved in DMSO (Sigma-Aldrich, USA) to a stock concentration of 10 mmol/L and stored at −80 °C. Puromycin (Sigma-Aldrich, USA) was dissolved in saline to a stock concentration of 1 mg/mL and stored at −20 °C. Leptomycin B (LMB, MedChemExpress, USA, 87081-35-4) was dissolved in DMSO (Sigma-Aldrich, USA) to a stock concentration of 10 ng/mL and stored at −80 °C.

### MiRNAscope

A tissue microarray (TMA, Shanghai Outdo Biotech, China) composed of 90 paired LUSC and adjacent normal tissues was employed in a miRNAscope assay (#324500, Advanced Cell Diagnostics, USA) to evaluate the expression of miR-375. The detailed clinical and pathologic information are listed in Supplementary Table S1. The method of miRNAscope analysis was described previously [11].

### Bioinformatic analysis

A LUSC dataset consisting of 502 tumor tissues and 49 normal adjacent tissues was obtained and preprocessed from the TCGA database (https://tcga-data.nci.nih.gov/tcga/)_._ MiRNA targets were predicted using publicly available algorithms, including RNA22V2 (https://cm.jefferson.edu/rna22/Interactive/), miRTarBase (https://mirtarbase.cuhk.edu.cn/), miRststem (http://mirsystem.cgm.ntu.edu.tw/), TargetScan (http://www.targetscan.org/vert_72/) and miRDB (http://mirdb.org/). A network of interactions was constructed using the STRING database (https://cn.string-db.org/), and STRING interaction genes were determined with GO and KEGG analyses using R software (version 3.6.3). GSEA was performed as described previously to test differentially expressed genes relevant to the expression of UBE3A in the TCGA-LUSC database [11].

### RNA sequencing

RNA sequencing was performed to determine the differentially expressed genes as well as the potential target genes of miR-375 when miR-375 was overexpressed in SK-MES-1 cells using the Illumina sequencing platform. The differentially expressed targets were defined by a threshold of *P* value < 0.01 and fold change >1.5 and then used for GO and KEGG analysis. Then, bioinformatics analysis was conducted according to the differentially expressed genes, which are listed in Supplementary Table S2.

### Western blot

Cells and tissues were lysed with RIPA lysis buffer (Boster, China, AR0102-100) with 1% protease inhibitors (Boster, China) for 30 min on ice. A total of 25 μg of protein was separated on 10% or 12.5% SDS-polyacrylamide gels, transferred to polyvinylidene difluoride (PVDF) membranes (Roche, Switzerland, 03010040001), and subsequently immunoblotted with the indicated antibodies, which are listed in Supplementary Table S3. An ECL kit (Beijing Applygen Technologies, Inc., China, P1010) was employed to visualize the targets.

### Quantitative real-time polymerase chain reaction assay (qRT‒PCR)

TRIzol reagent (Invitrogen Life Technologies, Carlsbad, USA) was utilized to extract total RNA from tissues and cells. Reverse transcription was performed with a ReverTra Ace qPCR RT Kit (TOYOBO, Japan). QRT‒PCR was carried out using TransStart^R^ Top Green qPCR SuperMix (TransGen Biotech, Beijing, China) on a StepOnePlus Real-Time PCR system (Applied Biosystems, USA). Expression of GAPDH was used as the endogenous reference for mRNA and U6 for the expression of miR-375. The primer sequences are listed in Supplementary Table S4. Each assay was repeated three times.

### Dual-luciferase reporter gene assay

A dual-luciferase reporter gene assay was performed as described previously [11].

### Cell transfection

Generation of stable miR-375 overexpression and knockdown cell lines were constructed as described previously [11].

For the rescue experiment, miR-375-overexpressing cells were transfected with CMV-MCS-PGK-Puro-UBE3A or CMV-MCS-PGK-Puro-empty as a control, and miR-375-silenced cells were transfected with shUBE3A or its control by using jetPRIME®. Transfection efficiency was determined by qRT‒PCR and/or western blots, and the cells were used for subsequent experiments. The shRNA sequences are shown in Supplementary Table S5.

### Cell viability and proliferation assays

The Cell Counting Kit-8 (CCK-8, Sevenbio, Beijing, China) was utilized to assess cell viability following the guidance of the manufacturer. Selected cells were seeded in 96-well plates for 1 to 4 days, and then, CCK-8 solution was added at a 1:10 dilution into the cultures at 37 °C for 1 h. A microplate reader (Thermo, China, Multiskan FC) was used to detect the absorbance at 450 nm.

EdU incorporation was performed using a Cell-Light TM EdU Apollo 567 In Vitro kit (RiboBio, Guangzhou, China) according to the manufacturer’s protocol. Cells were seeded onto 96-well plates and cultured in medium with EdU (50 mmol/L) and then incubated for 2 h, followed by fixation, permeabilization, and EdU staining with Apollo 567 and Hoechst 33342. The proliferation rate was defined as the percentage of EdU-positive cells (red) to total cells (blue) using ImageJ software.

### Apoptosis analysis

Apoptosis was quantified with the Annexin V-FITC Apoptosis Detection Kit (Dojindo, Japan). Briefly, harvested cells were resuspended in staining buffer at 1 × 10^6^ cells/ml. Then, 100 μl of the solution to a tube was transferred and incubated with 5 µL of Annexin V-FITC as well as 5 µL of PI for 15 minutes at room temperature, shielded from light. Apoptotic cells were determined by flow cytometry (BD FACSAriaTM II, New Jersey, USA).

### Cell migration and invasion assays

For the wound-healing assay, cells were seeded in six-well plates until cell confluence reached approximately 100% and then scratched with 10 µl pipette tips to generate a wound. The scratch recovery was captured at 0 and 48 h, and the rate of closure was calculated with ImageJ software.

For the transwell assay, cells (5 × 10^4^) were seeded in serum-free medium in the upper chamber (8-μm pore size, Corning, USA) without or with Matrigel (BD Biosciences) and incubated in a 24-well plate containing 600 µl of medium with 10% FBS for 24 h (for the cell migration assays) or 48 h (for the cell invasion assays). The cells on the lower surface of the membrane were then fixed with 4% paraformaldehyde and stained with crystal violet. Cell counts are expressed as the average number of cells per field of view.

### Immunoprecipitation (IP) analysis

Five micrograms of the appropriate primary antibodies or IgG was incubated with 30 μl of Protein A/G agarose beads (MedChemExpress, USA, HY-K0202) for 1 h at room temperature, followed by incubation with cell lysate in RIPA buffer containing PMSF for 2 h at 4 °C. The mixture was centrifuged, the pellet was washed, and the bead-binding proteins were eluted with 1× SDS buffer. Western blot analysis was performed using immunoprecipitated proteins.

### Immunofluorescence (IF) assay

Cells on the confocal dish were fixed with 4% paraformaldehyde, permeabilized, blocked and incubated overnight at 4 °C with primary antibodies against UBE3A and DUSP1. After PBS washes, the cells were incubated with a fluorescence-conjugated secondary antibody and mounted with DAPI. A confocal laser-scanning microscope was used for visualization.

### Cycloheximide (CHX) chase

Cells with overexpression or silencing of miR-375, UBE3A or vector were treated with 100 μg/mL cycloheximide (MedChemExpress, 66-81-9, USA) for 0, 1, 2, 4, 8, or 12 h to block protein synthesis. The indicated protein levels were examined by western blots.

### Ubiquitination assay

Stable cell lines with overexpression or knockdown of miR-375 were transfected with Flag-Ub vector, or cells were cotransfected with Flag-Ub vector and UBE3A expression vector or UBE3A shRNA for 36 h. Cells were then treated with MG132 (10 μM) for 12 h to inhibit proteasomal degradation, and the levels of DUSP1 ubiquitination were determined by IP followed by western blot assays with an anti-Flag antibody.

### Cytoplasmic and nuclear fractionation

Nuclear and cytoplasmic fractionation was performed with the Nuclear/Cytosol Fractionation Kit (Beyotime, China) under the manufacturer’s instructions. In brief, after the cells were washed and centrifuged, cytoplasmic extraction reagent A was added into each cell sample, vortexed for 5 s, rested on ice for 10–15 min followed by the addition of cytoplasmic extraction reagent B. After a short vigorous vortex, the cell lysates were kept on ice for 1 minute and spun for 5 min at 12,000×*g* at 4 °C. The supernatant was collected as the cytoplasmic fraction. The leftover was then resuspended in nuclear extraction buffer on ice for 30 min and spun for 10 min at 12,000×*g* at 4 °C. The supernatant was collected as nuclear extract.

### In vivo tumorigenesis assays

BALB/c nude mice (6 weeks old, female) were obtained from Beijing Vital River Laboratory Animal Technology Co., Ltd. Animals were randomly assigned to groups. For subcutaneous xenograft models, 1 × 10^7^ H2170-NC /H2170-sp miR-375-transduced cells or 1 × 10^7^ SK-MES-1-Vector/SK-MES-1-miR-375-transduced cells suspended in 100 µl of PBS and Matrigel (1:1) were implanted subcutaneously into the dorsal flank of the mice (*n* = 5). Tumor size was measured weekly with caliper measurements of tumor areas ((width)^2^ × length)/2) for 4 weeks. The tissue sections were subjected to hematoxylin and eosin (H&E) staining or immunohistochemical (IHC) staining. The mice treatment and collection of data and analysis of RNA and protein samples, H&E and IHC was performed by different groups on coded samples. All experimental procedures were approved by the Institutional Animal Care and Use Committee of the Center of Harbin Medical University.

### IHC staining assay

The mouse tumor tissues and the TMA (Shanghai Outdo Biotech, China) composed of 90 paired LUSC and adjacent normal tissues were deparaffinized, rehydrated and placed in sodium citrate buffer for antigen retrieval and treated with hydrogen peroxide for 10 min. After being blocked with 5% normal goat serum, the tissue sections were incubated with HRP-conjugated primary antibodies at 4 °C overnight, followed by incubation with secondary antibody for 30 min at room temperature. The signal was visualized using 3,3′-diaminobenzidine (DAB) as the substrate. Then, the slides were counterstained with hematoxylin. The scoring criteria have been described previously [42].

### Statistical analysis

Normal distribution test and test of homogeneity of variances were analyzed. The data in statistical tests are conformed to a normal distribution and the variance is similar. All in vitro experiments were repeated three times, and data are shown as the mean ± SEM (standard error of the mean). Sample sizes were determined based on prior literature and best practices in the field. Student’s paired or unpaired *t* test or one-way ANOVA was used for the comparison of significant differences between groups with GraphPad Prism 8.0 software. Correlations between the UBE3A levels and miR-375 expression were analyzed with Spearman’s correlation analysis. Associations between target gene expression and clinicopathological features were analyzed by Fisher’s exact test or *t* test. The overall survival curve was constructed by using R software (version 3.6.3) and analyzed by the log-rank test. Univariate and multivariate Cox proportional hazards regression analyses were employed to identify the independent factors for prognosis. Differences were considered significant when *P < 0.05, **P < 0.01, ***P < 0.001, and ****P < 0.0001. ns: no significance.

## Supplementary information

Table S1

Table S2

Table S3

Table S4

Table S5

Table S6

Figure S1

Figure S2

Figure S3

Figure S4

Figure S5

Supplementary figure legends

## Data Availability

TCGA, STRING, RNA22V2, miRTarBase, miRststem, Targetscan, miRDB database were employed in this article. The other datasets supporting the conclusions of this article are included within the article and its supplementary which is available at cell death discovery’s website.
