# Supplementary material for: MicroRNA-375 restrains the progression of lung squamous cell carcinoma by modulating the ERK pathway via UBE3A-mediated DUSP1 degradation
Source: Cell Death Discov. 2023 Jun 29;9:199. doi: 10.1038/s41420-023-01499-7 (PMC10310764; doi:10.1038/s41420-023-01499-7)
Supplement: Supplementary file 3 — Table S3 [file 41420_2023_1499_MOESM3_ESM.docx]

Table S3. Protein antibodies utilized for western blot, IHC, IP and IF in this study.

| Antibody | | Host | Manufacturer | Product code |
| --- | --- | --- | --- | --- |
| BCL2  BAX  E-cadherin | Mouse  Rabbit  Rabbit | | Proteintech  Abmart  Proteintech | 60178-1-lg  T40051F  20874-1-AP |
| N-cadherin | Mouse | | Proteintech | 66219-1-lg |
| MMP2 | Mouse | | Proteintech | 66366-1-lg |
| MMP9 | Rabbit | | Proteintech | 10375-2-AP |
| GAPDH | Mouse | | Proteintech | 60004-1-lg |
| UBE3A | Rabbit | | Proteintech | 10344-1-AP |
| DUSP1(for IF and WB) | Mouse | | SANTA | Sc-373841 |
| DUSP1 (for IHC)  Flag  c-Myc | Rabbit  Mouse  Rabbit | | Abmart  Proteintech  Wanleibio | T56588  66008-4-Ig  WL01781 |
| Phospho-p44/42 MAPK (Erk1/2) (Thr202/Tyr204) (D13.14.4E) XP® Rabbit mAb | Rabbit | | Cell signaling TECHNOLOGY | #4370 |
| ERK1/2 Rabbit Recombinant mAb  Normal Rabbit lgG  Normal mouse IgG  Cy3-conjugated Affinipure Goat Anti-  Mouse IgG(H+L)  FITC-conjugated Affinipure Goat Anti-  Rabbit IgG(H+L)  Ki67  Goat anti-Rabbit IgG  HRP-conjugated Affinipure Goat Anti-Mouse IgG(H+L) | Rabbit  Rabbit  Mouse  Mouse  Rabbit  Rabbit  Rabbit  Mouse | | Bimake  Cell signaling TECHNOLOGY  SANTA  Proteintech  Proteintech  Proteintech  Proteintech  Proteintech | A5029  #2729  Sc-2025  SA00009-1  SA00003-2  27309-1-AP   B900210  SA00001-1 |
