# Supplementary material for: MicroRNA-375 restrains the progression of lung squamous cell carcinoma by modulating the ERK pathway via UBE3A-mediated DUSP1 degradation
Source: Cell Death Discov. 2023 Jun 29;9:199. doi: 10.1038/s41420-023-01499-7 (PMC10310764; doi:10.1038/s41420-023-01499-7)
Supplement: Supplementary file 4 — Table S4 [file 41420_2023_1499_MOESM4_ESM.docx]

Table S4. The information of primers used for real-time PCR.

| Gene name | Forward | Reverse |
| --- | --- | --- |
| miR-375 | 5’-TTTGTTCGTTCGGCTCGC-3’ | 5’-CAGTGCGTGTCGTGGAGT-3’ |
| U6 | 5’-CTCGCTTCGGCAGCACA-3’ | 5’-AACGCTTCACGAATTTGCGT-3’ |
| UBE3A | 5’-ACGACATTGAAGCTAGCCGAAT-3’ | 5’-TGGACAGGAAGCACAAAACTCA-3’ |
| GAPDH | 5’-CACCCACTCCTCCACCTTTGA-3’ | 5’-ACCACCCTGTTGCTGTAGCCA-3’ |
| DUSP1 | 5’- TGCTGGAGGAAGGGTGTTTG-3’ | 5’- ATGCTTCGCCTCTGCTTCAC-3’ |
