# Supplementary material for: MicroRNA-375 restrains the progression of lung squamous cell carcinoma by modulating the ERK pathway via UBE3A-mediated DUSP1 degradation
Source: Cell Death Discov. 2023 Jun 29;9:199. doi: 10.1038/s41420-023-01499-7 (PMC10310764; doi:10.1038/s41420-023-01499-7)
Supplement: Supplementary file 5 — Table S5 [file 41420_2023_1499_MOESM5_ESM.docx]

Table S5. Target sequences for UBE3A.

| Name | Category | Sequence |
| --- | --- | --- |
| ShNC | Top strand | GATCCGTTCTCCGAACGTGTCACGTAATTCAAGAGATTACGTGACACGTTCGGAGAATTTTTTC |
| ShNC | Bottom strand | AATTGAAAAAATTCTCCGAACGTGTCACGTAATCTCTTGAATTACGTGACACGTTCGGAGAACG |
| ShUBE3A#1 | Top strand | GATCCGAGTACTGGGTCTGGCTATTTACTCGAGTAAATAGCCAGACCCAGTACTTTTTTTG |
| ShUBE3A#1 | Bottom strand | AATTCAAAAAAAGTACTGGGTCTGGCTATTTACTCGAGTAAATAGCCAGACCCAGTACTCG |
| ShUBE3A#2 | Top strand | GATCCGTCTGACTACATTCTCAATAAACTCGAGTTTATTGAGAATGTAGTCAGATTTTTTG |
| ShUBE3A#2 | Bottom strand | AATTCAAAAAATCTGACTACATTCTCAATAAACTCGAGTTTATTGAGAATGTAGTCAGACG |
| ShUBE3A#3 | Top strand | GATCCGACATCTCATACTTGCTTTAATCTCGAGATTAAAGCAAGTATGAGATGTTTTTTTG |
| ShUBE3A#3 | Bottom strand | AATTCAAAAAAACATCTCATACTTGCTTTAATCTCGAGATTAAAGCAAGTATGAGATGTCG |
