# Supplementary material for: MicroRNA-375 restrains the progression of lung squamous cell carcinoma by modulating the ERK pathway via UBE3A-mediated DUSP1 degradation
Source: Cell Death Discov. 2023 Jun 29;9:199. doi: 10.1038/s41420-023-01499-7 (PMC10310764; doi:10.1038/s41420-023-01499-7)
Supplement: Supplementary file 6 — Table S6 [file 41420_2023_1499_MOESM6_ESM.docx]

Table S6. GO and KEGG analysis of UBE3A and interaction gene.

| Ontology | ID | Description | GeneRatio | BgRatio | pvalue | p.adjust | qvalue |
| --- | --- | --- | --- | --- | --- | --- | --- |
| BP | GO:0043161 | proteasome-mediated ubiquitin-dependent protein catabolic process | 14/46 | 419/18670 | 8.27e-13 | 1.80e-09 | 1.05e-09 |
| BP | GO:0045088 | regulation of innate immune response | 12/46 | 452/18670 | 6.47e-10 | 1.20e-07 | 7.01e-08 |
| BP | GO:0002218 | activation of innate immune response | 10/46 | 319/18670 | 4.36e-09 | 5.28e-07 | 3.08e-07 |
| BP | GO:1901990 | regulation of mitotic cell cycle phase transition | 11/46 | 444/18670 | 7.67e-09 | 7.32e-07 | 4.27e-07 |
| BP | GO:1901987 | regulation of cell cycle phase transition | 11/46 | 480/18670 | 1.71e-08 | 1.38e-06 | 8.08e-07 |
| BP | GO:0031647 | regulation of protein stability | 8/46 | 284/18670 | 4.11e-07 | 2.04e-05 | 1.19e-05 |
| BP | GO:0050821 | protein stabilization | 6/46 | 178/18670 | 4.71e-06 | 1.09e-04 | 6.38e-05 |
| BP | GO:0060149 | negative regulation of posttranscriptional gene silencing | 3/46 | 19/18670 | 1.32e-05 | 2.34e-04 | 1.37e-04 |
| BP | GO:1901796 | regulation of signal transduction by p53 class mediator | 5/46 | 180/18670 | 7.84e-05 | 0.001 | 6.08e-04 |
| BP | GO:0050852 | T cell receptor signaling pathway | 5/46 | 202/18670 | 1.35e-04 | 0.002 | 9.52e-04 |
| CC | GO:0000502 | proteasome complex | 8/46 | 63/19717 | 1.63e-12 | 1.19e-10 | 5.86e-11 |
| CC | GO:1905369 | endopeptidase complex | 8/46 | 64/19717 | 1.85e-12 | 1.19e-10 | 5.86e-11 |
| CC | GO:0000790 | nuclear chromatin | 11/46 | 377/19717 | 7.91e-10 | 2.17e-08 | 1.07e-08 |
| CC | GO:0014069 | postsynaptic density | 7/46 | 324/19717 | 9.36e-06 | 8.99e-05 | 4.44e-05 |
| CC | GO:0032279 | asymmetric synapse | 7/46 | 328/19717 | 1.01e-05 | 9.27e-05 | 4.57e-05 |
| CC | GO:0099572 | postsynaptic specialization | 7/46 | 348/19717 | 1.49e-05 | 1.23e-04 | 6.09e-05 |
| CC | GO:0098984 | neuron to neuron synapse | 7/46 | 350/19717 | 1.54e-05 | 1.23e-04 | 6.09e-05 |
| CC | GO:0000151 | ubiquitin ligase complex | 6/46 | 282/19717 | 4.70e-05 | 3.22e-04 | 1.59e-04 |
| CC | GO:1904949 | ATPase complex | 4/46 | 101/19717 | 8.97e-05 | 5.56e-04 | 2.74e-04 |
| CC | GO:0070160 | tight junction | 4/46 | 128/19717 | 2.24e-04 | 0.001 | 5.73e-04 |
| MF | GO:0031625 | ubiquitin protein ligase binding | 11/46 | 290/17697 | 1.52e-10 | 2.69e-08 | 1.27e-08 |
| MF | GO:0061631 | ubiquitin conjugating enzyme activity | 6/46 | 38/17697 | 5.70e-10 | 3.55e-08 | 1.68e-08 |
| MF | GO:0004842 | ubiquitin-protein transferase activity | 10/46 | 382/17697 | 3.98e-08 | 8.28e-07 | 3.91e-07 |
| MF | GO:0019787 | ubiquitin-like protein transferase activity | 10/46 | 407/17697 | 7.22e-08 | 1.35e-06 | 6.38e-07 |
| MF | GO:0001085 | RNA polymerase II transcription factor binding | 6/46 | 155/17697 | 2.87e-06 | 3.36e-05 | 1.59e-05 |
| MF | GO:0070628 | proteasome binding | 3/46 | 17/17697 | 1.09e-05 | 1.02e-04 | 4.82e-05 |
| MF | GO:0051059 | NF-kappaB binding | 3/46 | 29/17697 | 5.73e-05 | 4.46e-04 | 2.11e-04 |
| MF | GO:0033613 | activating transcription factor binding | 4/46 | 85/17697 | 6.93e-05 | 5.19e-04 | 2.45e-04 |
| MF | GO:0019902 | phosphatase binding | 5/46 | 185/17697 | 1.15e-04 | 7.38e-04 | 3.49e-04 |
| MF | GO:0031072 | heat shock protein binding | 4/46 | 119/17697 | 2.55e-04 | 0.001 | 6.09e-04 |
| KEGG | hsa05165 | Human papillomavirus infection | 16/42 | 331/8076 | 2.80e-12 | 3.02e-10 | 1.77e-10 |
| KEGG | hsa04120 | Ubiquitin mediated proteolysis | 8/42 | 140/8076 | 4.79e-07 | 1.29e-05 | 7.57e-06 |
| KEGG | hsa03050 | Proteasome | 5/42 | 46/8076 | 3.49e-06 | 7.53e-05 | 4.40e-05 |
| KEGG | hsa05016 | Huntington disease | 9/42 | 306/8076 | 2.12e-05 | 3.81e-04 | 2.23e-04 |
| KEGG | hsa04530 | Tight junction | 6/42 | 169/8076 | 2.16e-04 | 0.002 | 0.001 |
| KEGG | hsa05022 | Pathways of neurodegeneration - multiple diseases | 8/42 | 475/8076 | 0.003 | 0.013 | 0.008 |
| KEGG | hsa05202 | Transcriptional misregulation in cancer | 5/42 | 192/8076 | 0.003 | 0.014 | 0.008 |
| KEGG | hsa04110 | Cell cycle | 4/42 | 124/8076 | 0.004 | 0.017 | 0.010 |
| KEGG | hsa04115 | p53 signaling pathway | 3/42 | 73/8076 | 0.006 | 0.024 | 0.014 |
| KEGG | hsa05010 | Alzheimer disease | 6/42 | 369/8076 | 0.011 | 0.035 | 0.021 |
