# Supplementary figures and images for: MicroRNA-375 restrains the progression of lung squamous cell carcinoma by modulating the ERK pathway via UBE3A-mediated DUSP1 degradation
Source: Cell Death Discov. 2023 Jun 29;9:199. doi: 10.1038/s41420-023-01499-7 (PMC10310764; doi:10.1038/s41420-023-01499-7)

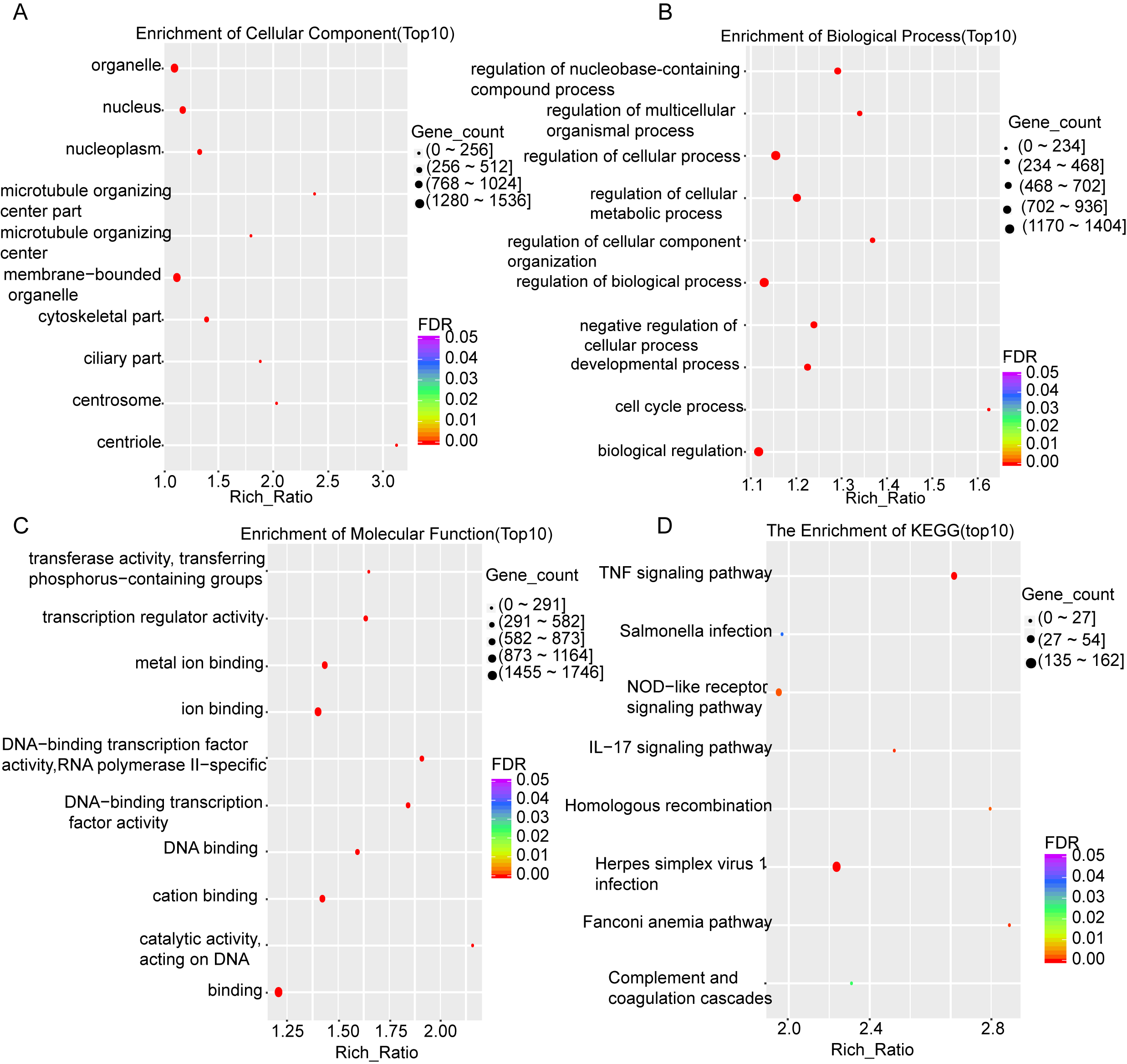

Supplement: Supplementary file 7 — Figure S1 [file 41420_2023_1499_MOESM7_ESM.tif]

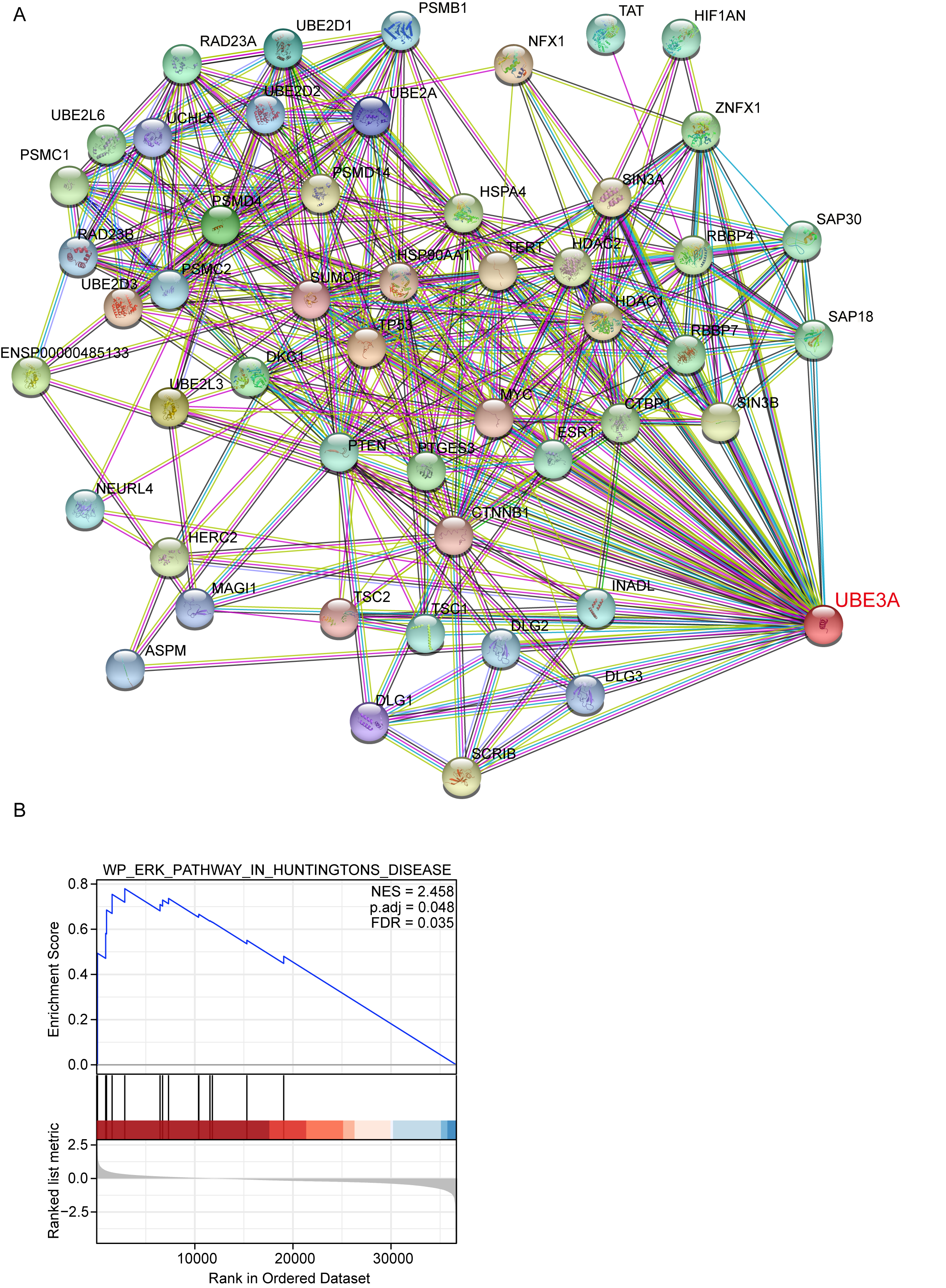

Supplement: Supplementary file 8 — Figure S2 [file 41420_2023_1499_MOESM8_ESM.tif]

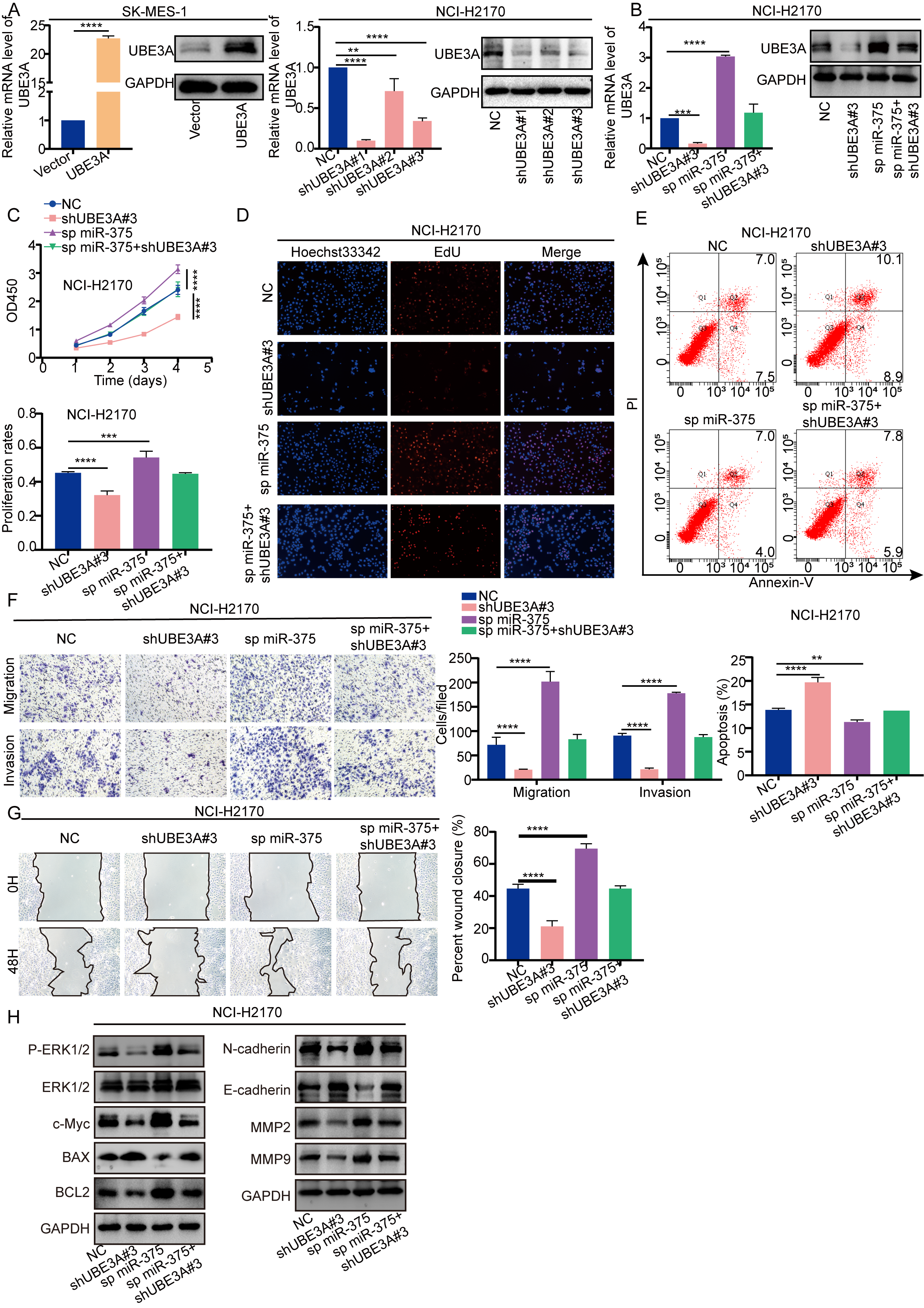

Supplement: Supplementary file 9 — Figure S3 [file 41420_2023_1499_MOESM9_ESM.tif]

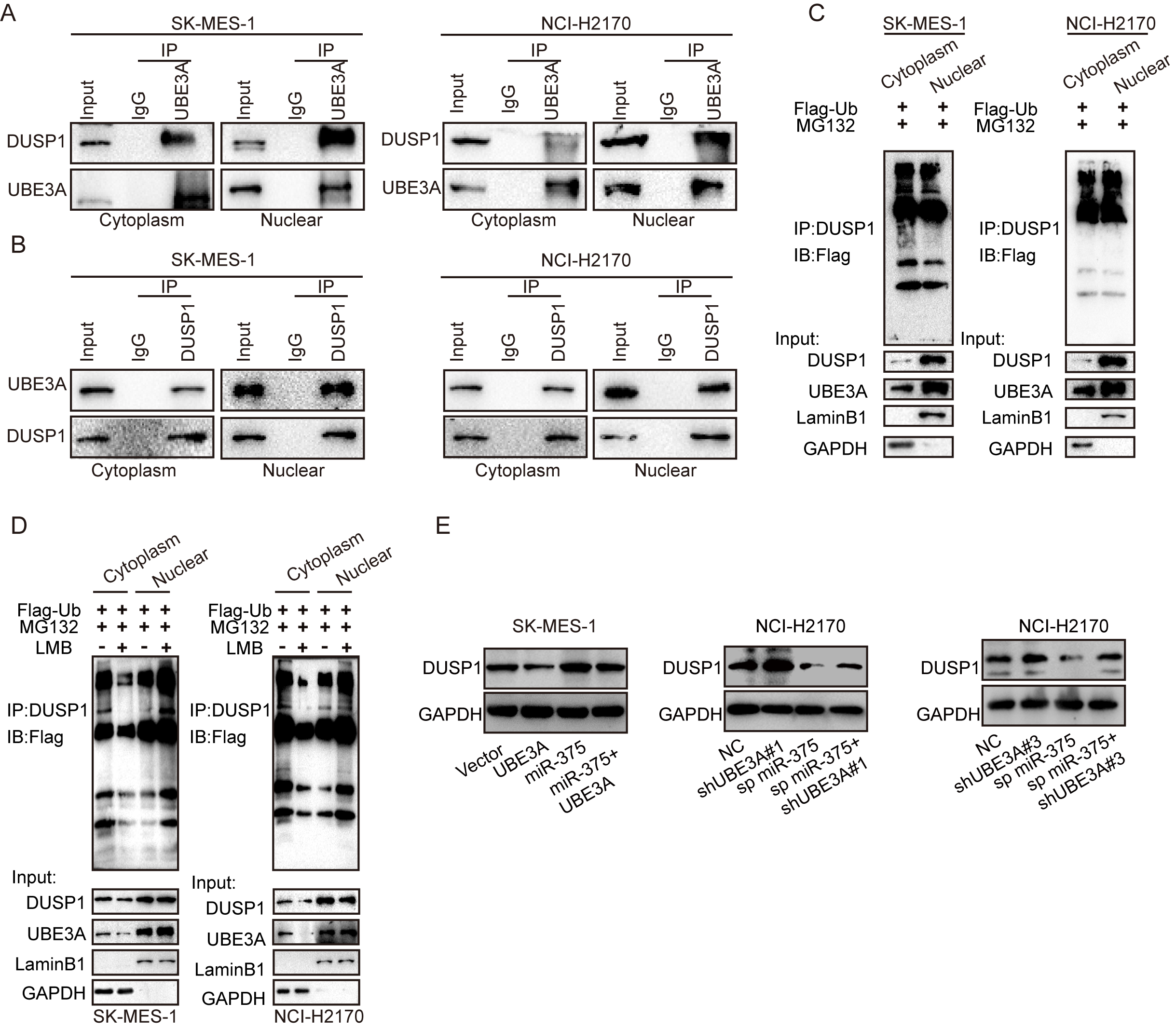

Supplement: Supplementary file 10 — Figure S4 [file 41420_2023_1499_MOESM10_ESM.tif]

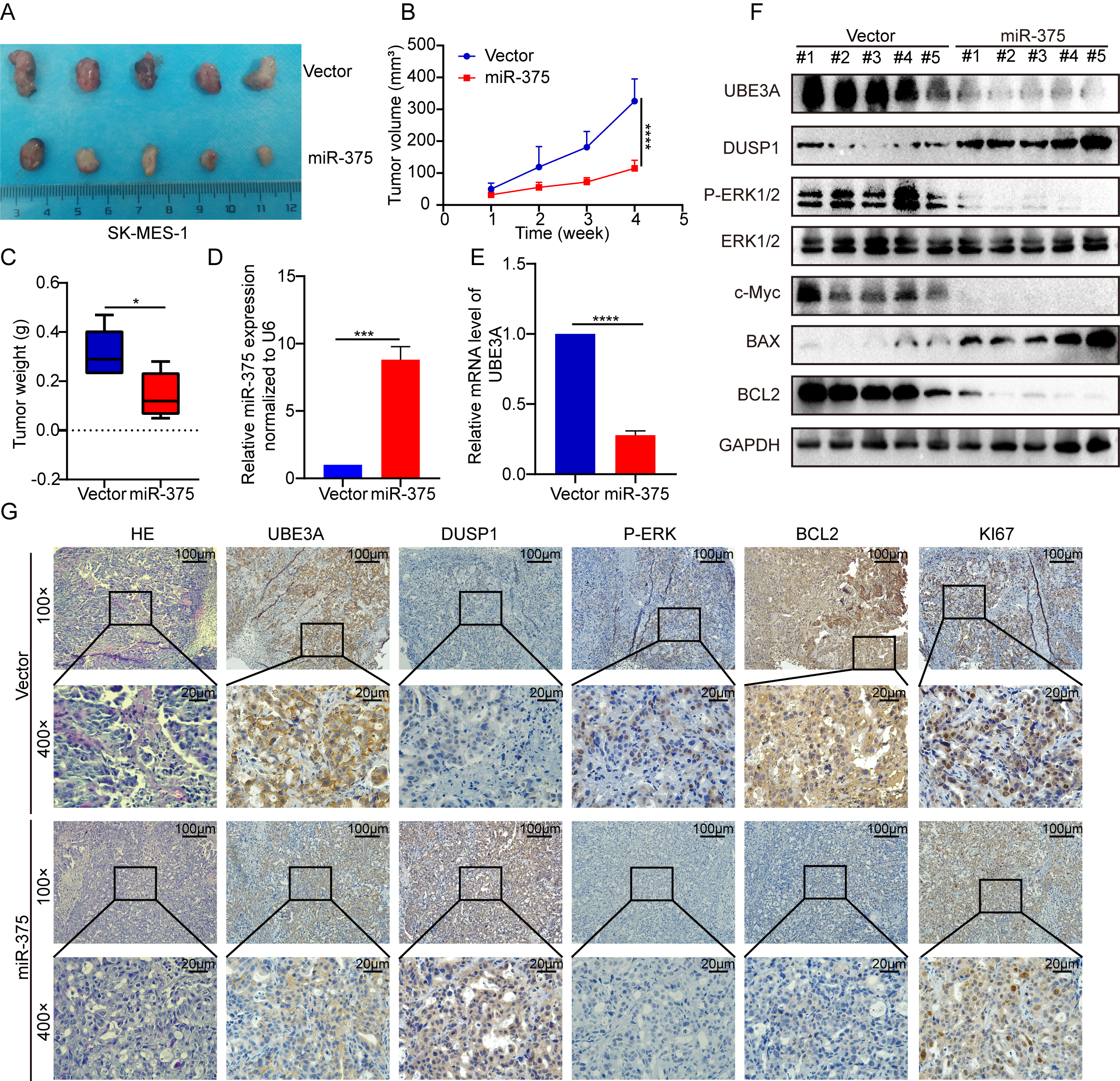

Supplement: Supplementary file 11 — Figure S5 [file 41420_2023_1499_MOESM11_ESM.tif]
