# Supplementary material for: MicroRNA-375 restrains the progression of lung squamous cell carcinoma by modulating the ERK pathway via UBE3A-mediated DUSP1 degradation
Source: Cell Death Discov. 2023 Jun 29;9:199. doi: 10.1038/s41420-023-01499-7 (PMC10310764; doi:10.1038/s41420-023-01499-7)
Supplement: Supplementary file 12 — Supplementary figure legends [file 41420_2023_1499_MOESM12_ESM.docx]

Figure S1. GO and KEGG analysis of differentially expressed genes in response to overexpression of miR-375. (A) Cellular component. (B) Biological process. (C) Molecular function. (D) KEGG enrichment analysis.

Figure S2. Bioinformatic analysis of UBE3A and interacting genes. (A) The STRING database was used to predict the network of 50 proteins that might interact with UBE3A. UBE3A is marked in red. (B) GSEA enrichment plots of differentially expressed genes between the high- and low-UBE3A expression groups.

Figure S3. UBE3A is required for miR-375-mediated inhibition of the malignant phenotype of LUSC by inactivating the ERK pathway. (A) The efficiency of UBE3A overexpression in SK-MES-1 cells and inhibition in NCI-H2170, measured by qRT‒PCR and western blots. (B) qRT‒PCR and western blotting showed the mRNA expression and protein expression of UBE3A in NCI-H2170 cells transfected with shUBE3A#3 and/or sp miR-375 vector(s). (C) A CCK-8 assays were used to determine the viability of NCI-H2170 cells transfected with shUBE3A#3 and/or sp miR-375 vector(s) at the indicated time points. (D) The proliferation of NCI-H2170 cells transfected with shUBE3A#3 and/or sp miR-375 vector(s), determined by EdU assays with magnification of 100×. (E) Flow cytometry analyses of cell apoptosis in NCI-H2170 cells transfected with shUBE3A#3 and/or sp miR-375 vector(s). (F) Transwell assays and (G) wound healing assays were used to test the migration and invasion of NCI-H2170 cells in response to suppression of miR-375 and/or UBE3A, magnification 100×. (H) Protein expression of p-ERK1/2, BAX, BCL2, c-Myc, E-cadherin, N-cadherin, MMP2 and MMP9 was tested by western blots in NCI-H2170 cells transfected with shUBE3A#3 and/or sp miR-375 vector(s).

Figure S4. DUSP1 ubiquitination occured in both cytoplasm and nucleus. (A)-(B) Cytosolic and nuclear protein fractions isolated from SK-MES-1 and NCI-H2170 cells were subjected to immunoprecipitation with negative control IgG, anti-UBE3A(A) or anti-DUSP1 antibodies(B). The immunoprecipitates were then detected using the indicated antibodies. (C) SK-MES-1 and NCI-H2170 cells were transfected with the Flag-Ub. After 48 h, cells cytosolic and nuclear fractions were used to test the polyubiquitination level of DUSP1. The selected cells were treated with 10 µM MG132 for 12 h before being harvested. (D) SK-MES-1 and NCI-H2170 cells were transfected with Flag-Ub. After 48 h, cells cytosolic and nuclear fractions were used to test the polyubiquitination level of DUSP1. The selected cells were treated with 10 µM MG132 and with or without 10ng/mL LMB for 12 h before being harvested. (E) Protein expression of DUSP1 was tested in SK-MES-1 cells transfected with UBE3A and/or miR-375 expression vector(s) and in NCI-H2170 cells transfected with shUBE3A and/or sp miR-375 vector(s). GAPDH was used as a loading control.

Figure S5. MiR-375 overexprssion delays LUSC tumorigenesis in vivo. (A) Representative images of tumors, (B) tumor volume and (C) tumor weight in nude mice bearing SK-MES-1 with or without miR-375 overexpression (n=5). (D)-(E) The expression of miR-375 (D) and mRNA level of UBE3A (E) in xenograft tumors, determined with qRT-qPCR. (F) Western blots and (G) HE and IHC were employed to analyze the expression of the indicated antibodies.
